# The cognitive benefits of basketball training compared to a combined endurance and resistance training regimen: a four-month intervention study

**DOI:** 10.1038/s41598-023-32470-2

**Published:** 2023-07-10

**Authors:** Iker Madinabeitia-Cabrera, Francisco Alarcón-López, Luis J. Chirosa-Ríos, Ignacio Pelayo-Tejo, David Cárdenas-Vélez

**Affiliations:** 1 Department of Physical Education and Sport, Faculty of Sport Sciences, University of Granada, 18071 Granada, Spain; 2 Sport and Health University Research Institute (iMUDS), University of Granada, 18071 Granada, Spain; 3 Department of General and Specific Didactics, Faculty of Education, University of Alicante, 03690 Alicante, Spain

**Keywords:** Psychology, Human behaviour

## Abstract

Neurocognitive function, especially executive functioning, is positively associated with better fitness or higher levels of physical activity (PA). Previous research suggests that combined endurance and resistance (AER+R) training leads to greater improvements than training in either modality separately. Dynamic team sports with cognitive dimensions, such as basketball (BAS), may be an excellent context for improving cognition. This study compared the effects of following a four-month PA training program in BAS versus AER+R on executive functions along with a control group with low PA. Fifty participants completed the training period and were randomly distributed into three groups: BAS (16 participants), AER+R (18), and control (16). Participants in the BAS group showed improved inhibition and working memory and those in the AER+R group showed improved inhibition and cognitive flexibility, while inhibition deteriorated in the control group. There were significant differences between groups only in inhibition. It appears that following a four-month PA training program is enough to enhance executive functioning, and improvements in inhibition are more evident when the program includes an open sport such as BAS.

## Introduction

Physical activity (PA) has positive effects on cardiovascular and general health, and it can also protect the human brain and cognition. Both cross-sectional and prospective studies have shown that individuals with better fitness or higher levels of PA tend to have higher levels of neurocognitive function compared to inactive or sedentary people^1^. Several of these studies have observed the effects on the executive functions (EFs) involved in higher cognitive processes. For instance, studies with adolescents have shown a relationship between PA and better cognitive outcomes, presenting improvements in EFs^2^, while low levels of PA are closely related to impaired EFs^3^. However, when this relationship is tested in experimental studies, the results are not entirely congruent. Some meta-analyses have found positive effects for aerobic (AER) exercise interventions on EFs^4^, but reviews such as Young et al.^5^, have observed cases in which no effects were reported. Although it has been consistently shown that PA has a positive effect on neurocognition, many questions remain about the factors that trigger cognitive benefits.

According to Miyake et al.^6^, EFs are related to attentional processes in the brain. Three important (core) EFs^7^ are related to this attentional process: updating, or constantly observing the environment looking for essential information and quickly adding or deleting information in the working memory (WM); shifting, or the capacity to switch between different tasks or mental sets and use attention with cognitive flexibility (CF); and inhibition control (IC), which is the ability to deliberately override dominant or prepotent responses to certain stimuli. One can thus hypothesize explanations for how PA interventions improve EF—that is, through (1) regulation of neurotrophins, (2) an increase in blood flow and circulatory angiogenesis increase oxygen saturation, and (3) better information processing because of an increment in brain neurotransmitters^8^.

Research on influence of PA on brain function has been experimentally developed in two different ways based on intervention length. The first are studies that observe the instant effect of PA in cognition—that is, the acute effect. The second set of studies investigate the chronic effect of regularly engaging in PA over time, but this group is relatively small compared to studies on acute effects. More studies on the chronic cognitive effects of PA are thus necessary to check if both cross-sectional and acute results are similar in the long term. To the best of our knowledge, the results of studies in these two paradigms are not entirely congruent. According to Álvarez-Bueno et al.^9^, these incongruent results may be due to the lack of control in variables such as individual factors (e.g., age or gender); task-related factors (e.g., intensity, frequency, overall duration); and contextual factors. We therefore conducted a chronic effect study that tried to control these variables as much as possible through participant recruitment and training characteristics (see Methods), although the authors are aware of the difficulty of controlling all possible variables that may interfere with the results.

The PA training modality generally applied in longitudinal studies reporting positive effects has been either aerobic (AER) or resistance exercise (R). Both types of training have, however, been shown to improve performance in several cognitive functions, including attention, information processing, and memory^10^. Interestingly, it has been reported that the combination of AER and R exercises in a PA training program (AER+R) causes extensive overall improvements, rather separate improvements to attention capacity and concentration^8^. A meta-analysis conducted by Colcombe et al.^4^ also revealed that the most significant benefits to EFs were achieved when AER was paired with R.

Likewise, some evidence has shown that performing a PA in the presence of external stimuli, typically via game-like conditions that require information processing and working memory to perform successfully, can also contribute to improved EFs. The recent study by Muller et al.^11^ stated that PA interventions requiring constant cognitive and motor learning are more efficient in inducing cognitive benefits than the repetitive and cyclical activities generally used in AER and R studies. That is, rather than only performing a physical exercise, there could be more cognitive benefits if the exercise occurs in the context of a cognitively stimulating environment^12,13^. This combined dual-task training, in which the participant engages in PA and cognitive tasks simultaneously, is more cognitively demanding, because it involves additional cognitive processing to integrate and coordinate the two tasks at the same time^14^. The environmental conditions of the game (or task) places individuals in a continuous process of initiation, control, and flexibility to modify actions, which is believed to strengthen component processes of EF and memory storage^15^. The meta-analysis performed by Ludyga et al.^16^ with healthy adults highlighted that coordination exercises (with cognitive and attentional requirements) yielded the most significant effect on EFs.

Researchers thus consider that dynamic team sports, such as basketball (BAS), might be an excellent context for improving cognition^15^. The situations generated in this type of sport are complex due to their great dynamism, temporal restrictions, and the high number of stimuli requiring attention, which ensures mental commitment and thus stimulates the EFs^17^. The grade of uncertainty generated in this practice also produces considerable activation of the neural circuits and structures of the prefrontal cortex, which are related to EFs^18^. These sports demand that the athletes, besides putting forth a high degree of physical effort, also pay attention to the continually changing environment to perceive the information needed to make a decision (e.g., to observe the movements of teammates and opponents) and execute it^19,20^. These skills are directly linked with the EFs, and it is no surprise that elite players show better levels of EFs compared to amateur or sedentary individuals^21^. Neuroimaging studies have revealed that children who practice team sports show greater development in prefrontal areas because of their deeper information-processing requirements (Carey, Bhatt, & Nagpal^22^). Nevertheless, many studies in this area have been cross-sectional (i.e., comparing elite athletes at different levels with amateur or sedentary individuals). It could thus also be the case that individuals with better EFs are more likely to become skilled athletes (reverse causality), so it more longitudinal and experimental studies are necessary to clarify the benefits of practicing these sports.

To the best of our knowledge, comparing the longitudinal effects achieved in PA with cognitive demands versus without them (i.e., BAS versus AER+R) has not yet been studied in young adults. This study therefore compared the effects of following a four-month training program on the EFs—specifically IC, WM, and CF. Finally, to palliate any potential methodological problems that might lead to results incongruent with the literature, the entire study sample was composed of university students with similar levels of fitness, body composition, PA habits, dynamic team sport experience, and age; a control group was also included. We hypothesized that the BAS group, considering that the participants would engage in training with aerobic effort and attention to external stimuli, would show better EFs at the end of the intervention compared to individuals in the AER+R and CON groups.

## Methods

### Ethical clearance

Recruitment and experimental procedures for this study complied with the Declaration of Helsinki. Approval was granted by the Ethics Committee on Human Research of the University of Granada, Spain (419/CEIH/2017). All volunteers were informed about the experimental aims and conditions and signed an informed consent form before the study.

### Power analysis

To estimate the sample size, an a priori power calculation (G*Power version 3.1)^23^ was performed. First, according to the exercise and cognition literature^10,24,25^, the parameters applied were power = 0.95, α = 0.05 and effect size = 0.1 (small). However, the output of the minimum sample size was 390 participants, which would make it very complex to conduct the study. So, the effect size was changed to 0.25 (medium), which has been observed in general experiment studies as a reasonable estimation^26^; this yielded a minimum sample size of 60.

### Participants

**Figure 1:**
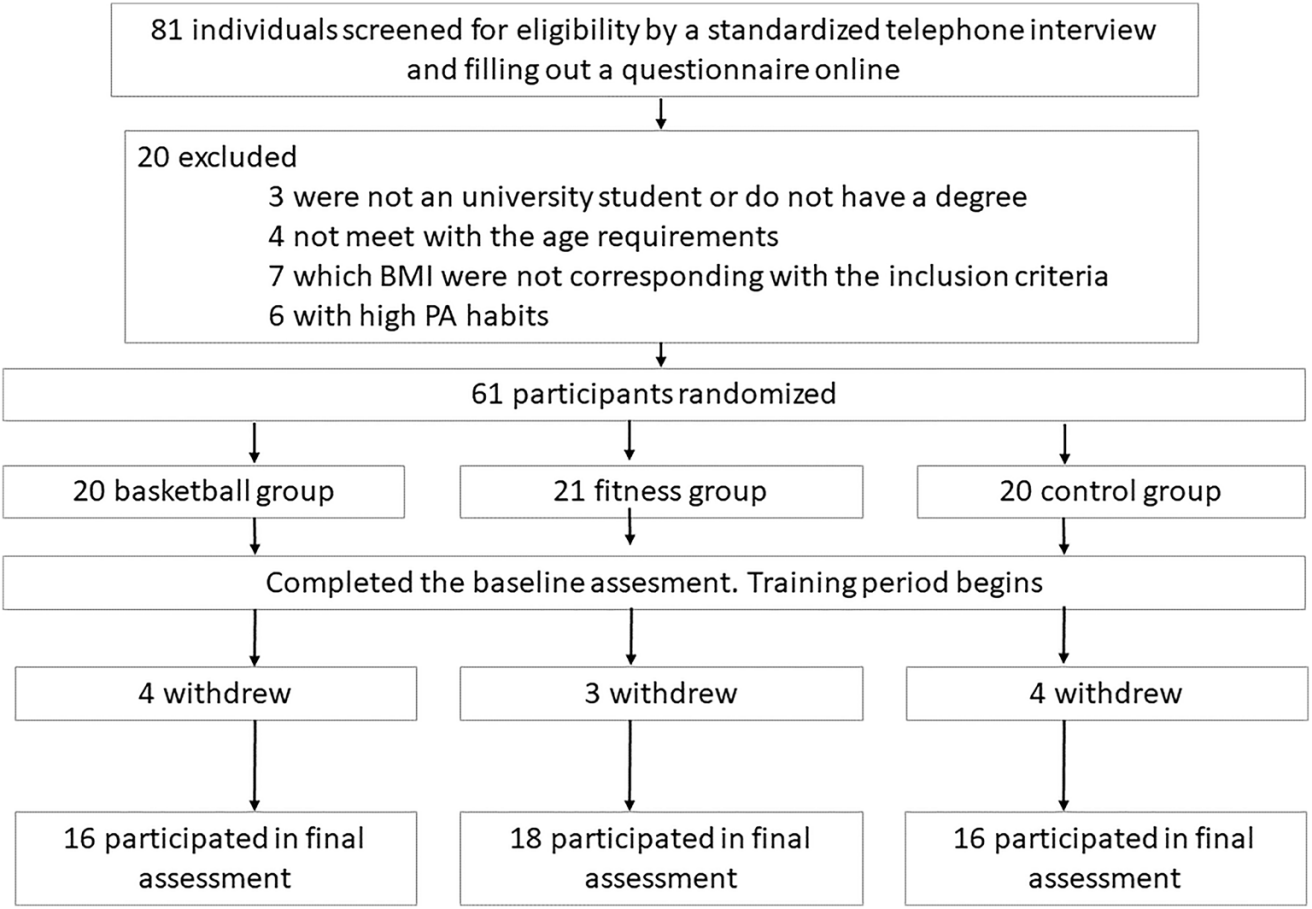
Flowchart of the participants of the study.

Eighty-one university students were recruited using an informative flyer and underwent screening by a standardized telephone interview and filling out an online questionnaire. The inclusion criteria established were: (1) aged 18–28 years old with a university degree or present university student status; (2) low PA habits (assessed by METs scale, see Variables section) and not having competed in a federated league for a dynamic team sport similar to basketball (e.g., football, handball, hockey); (3) BMI in the normal weight range; and (4) did not have a current medical condition for which exercise would be contraindicated. After applying the inclusion criteria, the number of participants was reduced to 61, of which 50 reached the end of the training period (see the flowchart in Fig. 1). The random distribution of those 50 participants was: BAS group, 16 participants (five women, mean age 24.19, *s* = 3.16); AER+R group 18 participants (eight women, mean age 23.72, *s* = 3.02); and a low-PA control group (CON), 16 participants (seven women, mean age 24.19, *s* = 2.99).

### Study design

A pre-post experimental study design was used to test the effects of the different PA programs on the EFs, along with the low PA control group. The two experimental groups followed a four-month PA training program of two hours per week. In one intervention, the participants followed a PA training with cognitive demands by playing basketball, while the other experimental group followed a regular fitness training program combining aerobic and resistance exercises.

## Variables

### PA habits

The International Physical Activity Questionnaire Short Form (IPAQ-SF; Lee, Macfarlane, Lam and Stewart^27^) is a 7-item questionnaire designed to measure respondents’ PA (e.g., Typically, how much time in total did you spend on intense physical activity on one of those days?). According to the answers, MET units are calculated: more than 600 METs indicate that the individual has a moderate PA habit and more than 3000 METs are considered a vigorous PA habit, while scores not reaching the moderate levels indicates a low PA habit (inclusion criteria).

### Endurance performance

Maximal incremental effort was performed to assess the endurance fitness level. It was carried out in an h/p/COSMOS pulsar (Nussdorf-Traunstein, Germany). The test began with a 3-min warm-up at 8 km·h^−1^, at 1% slope; the treadmill speed was set to 10 km·h^−1^, from which the incremental part of the test started. The treadmill speed was increased 0.25 km·h^−1^ every 15 s until volitional exhaustion. After exhaustion, an active recovery period was established consisting of participants walking at 4 km·h^−1^ (0° slope) for 5 min. Participants wore a fall prevention system during the entire session. Pulmonary gas exchange and EKG signal (Ultima CardiO2; Medical Graphics Corporation, St. Louis, USA) were continuously recorded in the whole process. The fitness measure was VO2max (oxygen consumption at exhaustion). The test was carried out under the control of a doctor in sports medicine.

### Resistance performance

The DynaSystem Research Functional Dynamometer (SYMOTECH, Granada, Spain) was used to evaluate the resistance performance by calculating the capacity to generate isometric strength in both the lower trunk (i.e., quadriceps) and upper trunk (i.e., biceps), following the evaluation procedure described below.

### Split squat

While standing upright on the DynaSystem Research Functional Dynamometer, the subject did a split squat and hyperextended the hip of the non-dominant leg, placing the superior aspect of the foot on a stable bench of approximately 75 cm in height. The non-dominant leg was placed on the bench at approximately 90° of femorotibial flexion and slight hip hyperextension. The knee of the dominant leg was exactly placed at 65°, positioning the foot above the DynaSystem Research Functional Dynamometer. An expert staff member used a universal goniometer to measure the angle of joint motion following the current guidelines^28^. The stance width was horizontally measured from the heel of the lead foot to the edge of the bench where the superior aspect of the foot was placed. At this point, subjects were asked to maintain a neutral spine, chest, and head position while facing forward. After obtaining balance and composure with both hands separated at shoulder width and resting on the wall at face height (i.e., maintaining proper posture), the subjects performed an 8 s maximum isometric split squat. This procedure has been used in other studies^28^.

### Biceps isometric strength

Participants stood on top of the machine. The foot opposite to the arm executing the gesture was placed parallel to the cord, while the other foot was placed behind. Both feet were placed on the line marked by the width of the shoulders. The knees were semi-flexed at approximately 15° (0° = full knee extension), the back straight, the shoulders aligned, and the gaze always straight ahead. The elbow flexion angulation was 45° or 90° (0° = full elbow extension).

### Intelligence level

The matrix reasoning test from the WAIS-III battery^29^ was used to assess the approximate IQ level of the participants, which was used as a control measure for the EF results. It consists of a non-verbal intelligence test in which a total of 26 figures with one portion missed are presented to the participant consecutively. From a total of five options, the participant has to answer which of them is more suitable to fill the gap with no time limit. This test can be used for different cultural and socioeconomic groups to capture general intelligence.

### Inhibition control

A Spanish adaptation of Golden’s Stroop test^30^ was used to evaluate IC (i.e., the inhibition response). This test consists of a total of three different conditions, which are similar to each other in the number of stimuli (i.e., 100 stimuli) printed on a sheet of paper in which the participants have 45 s to determine the maximum correct answers. The three conditions are: (1) read the words blue, red, and green that are printed in black; (2) 100 lines with the text “xxx” printed in different colors which the participants have to recognize; (3) the word written do not have the same color ink, and the participant has to name the color ink (i.e., “red” is printed in blue, participant has to answer blue). Correct responses and the index Stroop interference score were calculated for each participant.

The flanker task^31^ was also applied to assess IC (i.e., perceptual inhibition). An array of five black arrows was presented centrally on the white background of a 15.6″ computer screen. Participants, who were seated, were instructed to respond as quickly as possible to the directionality of the central target arrow using a computer mouse: right-click if the central arrow is pointed to the right, left-click if it is pointed to the left. Congruency was varied by manipulating the directionality of the flanking arrows. Flanking stimuli were randomly presented and could be either congruent (i.e., all arrows facing in the same direction, “< < < < <” or “> > > > >”) incongruent (i.e., flanking arrows facing the opposite direction, “< < > < <” or “> > < > >”) or neutral (i.e., only the central arrow appears, “·· < ··” or “·· > ··”). Stimuli were presented for 100 ms with a variable inter-stimulus interval of either 900, 1100, or 1300 ms. Participants completed 36 practice trials before completing two blocks of 144 trials, resulting in a task that took no more than 15 min to complete.

### Working memory

Participants performed the Letters and Numbers test from the Wechsler Adult Intelligence Scale-3 cognitive test battery (WAIS-III; Wechsler, 2006). The test consists of an instructor telling the participant, at a slow rhythm in a loud and clear voice, an alternate sequence of letters and numbers; the participant must first order the block of numbers from least to greatest and then the block of letters in alphabetical order (i.e., if the instructor says “B-5-3,” the participant has to answer “3-5-B”). The test begins with a chain of three elements, to which an additional element is added every three tests. The test ends once the participant has not completed at least one test of the three that form part of each chain. Each test performed correctly is scored with one point.

### Cognitive flexibility

The Trail Making Test^33^ was used to calculate CF. It consists of two parts: (1) a sheet of paper on which the numbers from 1 to 16 are distributed, and the participant has to draw a line that unites the numbers from low to high as fast as possible; (2) a sheet of paper on which numbers (from 1 to 16) and letters (from A to P) are distributed, and the participant has to draw a line following a sequence of uniting the numbers from low to high and letters alphabetically alternatively (i.e., 1-A-2-B-3-C). The variable of interest was calculated by the differential score of the time to perform part 2 minus the time to perform part 1. In case of a mistake during the participant’s performance, the instructor immediately indicates that there was an error and the participant had to go back to the previous step.

### Procedure

First, there was a meeting with all of the participants to explain the study and familiarize them with the cognitive tests. Participants signed their consent forms and were assigned to a group. The pre-evaluation was assessed before beginning the intervention in one week following a counterbalanced order distributed in three blocks, with 48 h of separation: endurance, resistance, and EFs/intellectual performance (the order was also counterbalanced with a 3-min rest between tests). The participants followed a four-month training with two one-hour sessions per week. Two of the groups were experimental (BAS and AER+R), while the last was a control group (CON). When the intervention was completed, the three groups performed the post-evaluation tests in the same order as in the pre-evaluation, although intellectual performance was not assessed, because it was used only to check that there were no differences in general intelligence between them. The procedure of each group was the following:

### BAS group

Although playing BAS requires individuals to play in situations with cognitive demands, we were afraid that the few hours per week might not be enough to cause chronic effects if the intervention consisted only in free-game situations (e.g., matches following the sport rules), and there could be a risk that the participants were more focused on the motor demands of execution (e.g., passing, receiving, dribbling), rather than on the tactical demands, which would result in inadequate cognitive stimulation. Thus, the authors considered dividing every session for the BAS group into three blocks: (1) a passing game; (2) the development of individual skills with a direct opponent; and (3) a collective game with specific rules. The order of these three blocks was changed in every session. Participants warmed up for five minutes individually before performing the blocks. Task difficulty increased according to the level of proficiency demonstrated.

In the passing game block, the participants were divided into two teams. Each team had a ball, and the game consisted of one team having to use their ball to touch the participant of the other team who has the ball, while the other team has to perform ten passes. The general rules were that the participant with the ball cannot move, so the team had to cooperate and make good passes to achieve the goal, and every time a participant passed, it was mandatory to move to another place, so staying in the same place was forbidden. The difficulty progressively increased over the training period by adding more rules such as not repeating the pass to the same person who did the previous pass, not looking at the person who is going to receive the ball, the type of pass has to be different from the previous one, a reduction in the game space, the presence of a third ball on the floor which the team that is trying not to be tagged must roll with their feet, and the team trying not to be tagged was assigned to wear two different colors and the ball could not be passed to a teammate wearing the same color.

The development of individual skills with a direct opponent consisted of tasks in which participants learned individual basketball technical skills (e.g., dribbling, shooting) with an ever-present opponent trying to grab their ball. Specific rules that required them to use their attention capacity included making it progressively more demanding, such as changing the dribbling hand, passing the ball to the instructor when he raised his hand, ending in a shoot in a one-on-one situation if the instructor showed a yellow color and in a lay-up if the card was red. Non-compliance with these rules led to an attacker-defender role change.

Respecting the collective game, the specific rules consisted of tasks somewhat similar to official basketball matches but applying task constraints, such as a time possession limit of 10 s, limiting the number of passes, the inclusion of another teammate, and playing in offensive or defensive advantage.

Finally, at the end of every session, all of the participants had to report their perceived effort according to the RPE scale.

### AER+R group

In each session, the AER+R group performed alternative blocks of aerobic and resistance training, and the sum of those blocks resulted in 50% aerobic training plus 50% resistance training. Participants performed a 5-min warm-up before the beginning of the session. The aerobic training program followed a HIIT protocol: four bouts of 3 min at 85–95% HRmax (obtained in the maximal endurance test) interspersed and 5 min of active recovery at 75–85% HRmax. Resistance training consisted of a combination of isometric training and 12–15 repetitions at 50%–70% of the maximum isometric strengths obtained in the DynaSystem Research Functional Dynamometer, distributing all of the major muscle groups between the two sessions of the week.

Like the BAS group, all of the participants answered the RPE scale when the session was concluded. This information was used to increase or decrease the intensity of the training individually.

### Control group

Considering that the participants fulfilled the PA habits requirements to participate in the study, they were asked to maintain the same activity during the following four months. The researchers periodically contacted these participants (2–3 times per month) to ensure that they were maintaining the same schedule.

### Statistical analysis

Data summaries were computed for the whole sample. First, a Shapiro–Wilk normality test was conducted for all of the variables of interest. Second, to check that before the intervention there were no differences between groups in terms of individual factors, pre-intervention variables (see points from 2.5.2 to 2.5.6 in the methods section) were submitted, according to their normality data, to their respective statistical analysis (i.e. ANOVA or Kruskal–Wallis), showing that there were no significant differences between groups. Again, PA habits were not analyzed because they were only used as inclusion criteria. Third, to check that the training program caused benefits in fitness, given that certain variables exhibited a normal distribution while others did not, suggesting the need to employ distinct statistical methods for each variable, and after ensuring that no significant differences were present at the outset, a differential score (Δ; post minus pre score) was computed for both VO2max and strength variables. This score was then subjected to both a paired sample t-test and the Wilcoxon test, based on the normality of their respective datasets. Fourth, as in the previous step, to observe the group effect in the EFs, ΔWM, ΔCF, and ΔIC were submitted to a paired sample t-test and Wilcoxon test. Finally, to establish significant differences in the variation of EFs between groups, ΔWM, ΔCF, and ΔIC were submitted to ANOVA and Kruskal–Wallis tests.

The significance level was set at 0.05, and Bonferroni correction for multiple comparisons was used where applicable. The standardized effect size was reported employing the partial ƞp^2^ for Fs and *d* in post-hoc analysis and t-tests, and *r* for the Wilcoxon paired-sample test, following the formula z/√(n), where *z* is the z-statistic and *n* the number of observations. Partial ƞp^2^ was based on Cohen’s *f*, which defines small, medium, and large as, respectively, 0.10, 0.25, and 0.50, which corresponds to an ƞ^2^ of 0.0099, 0.0588, and 0.1379, and both *d* and *r* use Cohen’s interpretation guidelines of 0.1 (small effect), 0.3 (moderate effect), and above 0.5 as a strong effect. The JASP statistics package (version 0.8.1.2) was used for analysis.

## Results

Means and standard deviations for each variable of the study are displayed in Table 1. No significant differences between groups were observed in age, BMI, PA habits, intellectual level (i.e., matrix reasoning test), and both fitness and cognitive variables before the intervention. Moreover, after every session, all of the participants reported their rate of perceived effort (RPE). Thus, before the intervention, there were no differences between groups at cognitive and fitness levels, and both groups trained at the same intensity.

**Table 1:** Summary descriptive statistics for the variables of the study.

Variable	Basketball (n = 16; 5 women)		AER+R (n = 18, 8 women)		Control (n = 16; 7 women)	
	Mean	SD	Mean	SD	Mean	SD
Age	24.19	3.16	23.72	3.02	24.19	2.99
BMI	23.36	3.11	23.37	1.97	22.21	2.61
Matrix score	21.19	2.16	21.11	2.37	20.31	3.11
VO2 max PRE	44.48	4.99	37.72	9.36	40.39	6.91
VO2 max POST	48.125	4.54	44.70	8.61	40.34	7.73
Biceps strength PRE	15.39	5.12	13.41	5.32	13.51	5.45
Biceps strength POST	15.24	4.73	14.87	4.91	14.38	5.19
Quadriceps strength PRE	44.74	25.86	35.37	26.46	53.03	28.47
Quadriceps strength POST	64.32	23.95	60.28	22.78	65.68	28.58
Letters and numbers test PRE	11.38	2.87	12.39	2.06	11.13	1.89
Letters and numbers test POST	13.13	2.33	13.11	2.34	12.19	2.58
Trail making test PRE	31.38	28.58	35.22	29.68	29.94	12.08
Trail making test POST	30.13	12.24	23.06	8.32	30.94	14.53
Stroop part 3 score PRE	55.01	15.14	57.89	11.38	53.01	13.16
Stroop part 3 score POST	62.38	13.53	61.67	13.33	56.01	10.74
Stroop interference PRE	45.07	9.06	51.34	8.76	48.33	5.93
Stroop interference POST	50.83	6.01	53.49	8.11	49.61	4.97
Flanker congruent acc. PRE	99.22	1.67	99.51	1.13	99.33	1.47
Flanker congruent acc. POST	99.73	0.71	99.53	0.89	98.31	2.43
Flanker congruent rt. PRE	518.80	66.887	518.69	57.48	517.21	37.11
Flanker congruent rt. POST	494.39	74.01	500.85	61.55	504.09	47.25
Flanker incongruent acc. PRE	92.898	8.297	93.17	5.63	94.35	5.93
Flanker incongruent acc. POST	91.92	6.07	92.47	7.01	90.62	9.16
Flanker incongruent rt. PRE	606.82	70.81	610.86	64.39	625.11	78.84
Flanker incongruent rt. POST	573.79	85.59	586.49	71.69	599.19	60.01
Flanker neutral acc. PRE	99.35	1.24	99.17	1.71	99.34	2.11
Flanker neutral acc. POST	99.34	1.25	99.31	1.42	98.56	2.81
Flanker neutral rt. PRE	501.28	50.41	514.71	58.74	516.41	31.91
Flanker neutral rt. POST	484.01	60.52	490.75	57.91	494.79	37.73

*SD* Standard deviation, *AER+R* experimental group which follow a 4-month training program of aerobic and resistance exercises combined, *acc.* Accuracy, *rt.* reaction time.

### Manipulation check

**Figure 2:**
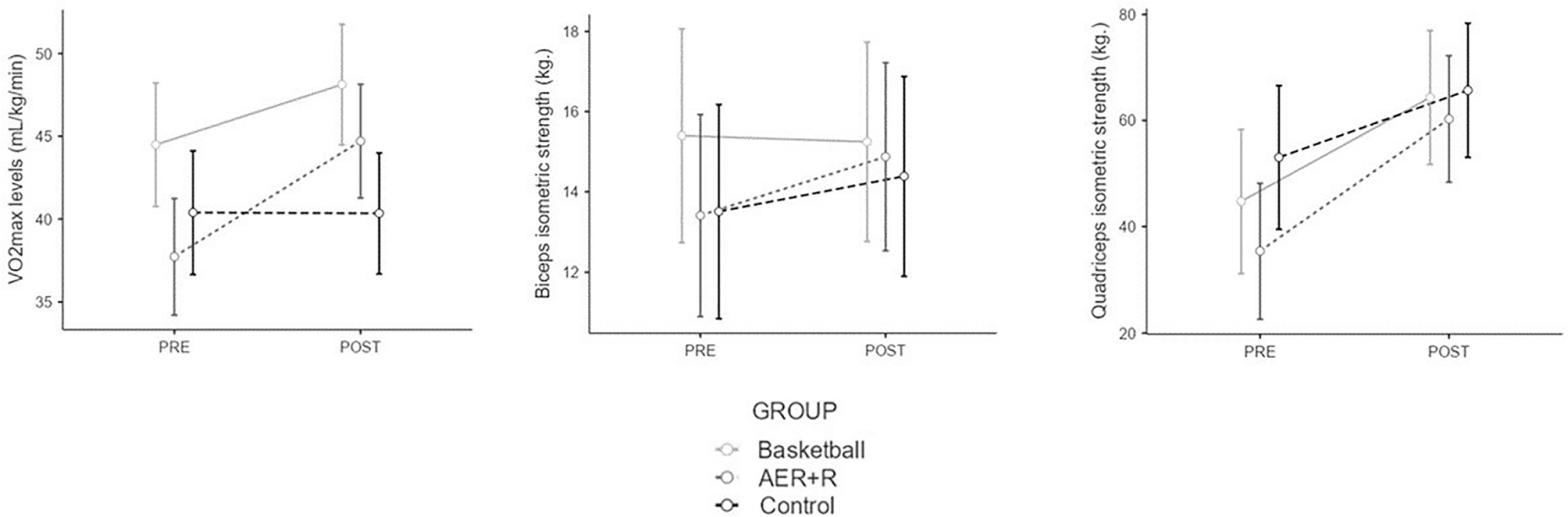
Physical condition manipulation check. Basketball group improves significantly in vo2 levels and strength in quadriceps. Fitness group (AER+R) improves in vo2 levels and strength in biceps and quadriceps. Control group did not obtain significant differences in any fitness test.

The results showed that BAS group improved significantly in VO2max levels (t =  − 2.874; *p*-value < 0.001; d =  − 1.25) and quadriceps strength (Z =  − 2.638; *p*-value = 0.008; r =  − 0.66). The AER+R group improved in both aerobic (t =  − 3.387; *p*-value = 0.004; d =  − 0.798) and biceps resistance levels (Z =  − 2.722; *p*-value = 0.006; r =  − 0.642) and quadriceps (Z =  − 3.682; *p*-value < 0.001; r =  − 0.868). The CON group did not show significant differences in any fitness test. This result is illustrated in Fig. 2.

### Group effect

**Figure 3:**
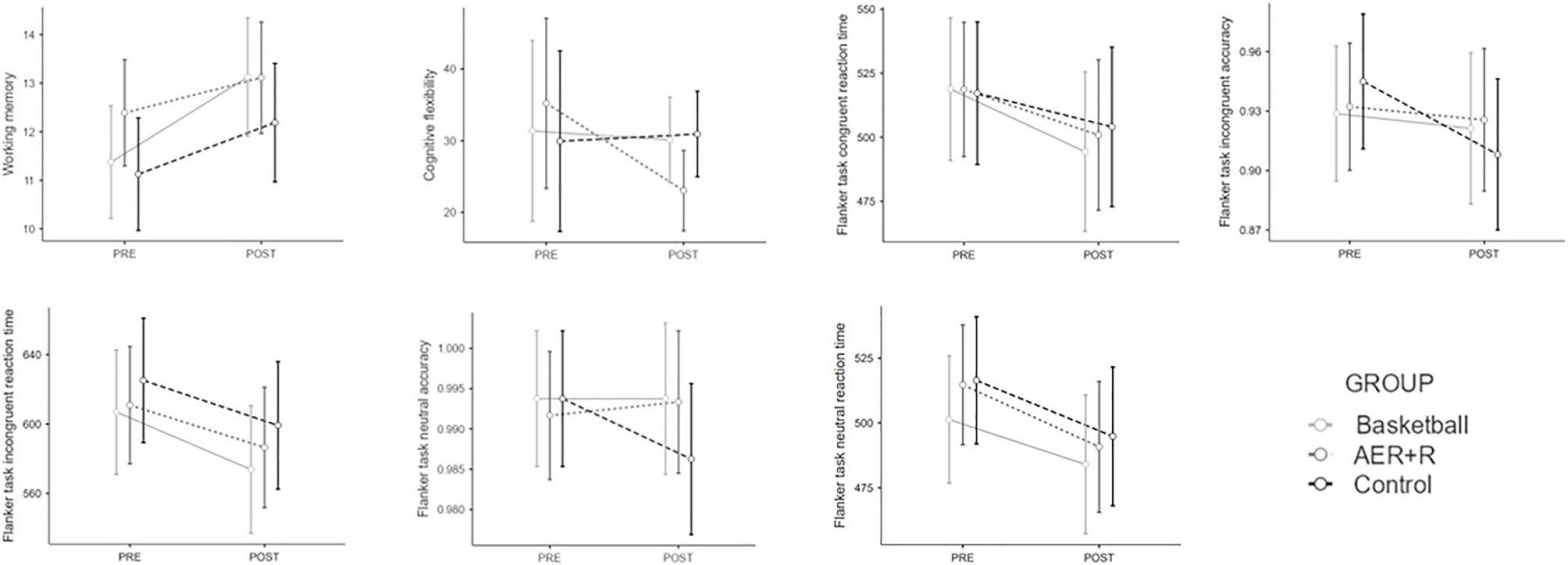
Group effects in executive functions. Basketball group significantly improved in working memory, Stroop part 3 and interference score, and the reaction time in flanker task incongruent part. Fitness group (AER+R) significantly improved in cognitive flexibility (note that in trail making test, less is better), and the reaction time in both congruent and incongruent parts of the flanker task. Control group significantly worsened in the accuracy of the flanker task in the congruent and incongruent trials but improved in the reaction time of the neutral part.

First, considering that each EF variable was analyzed for each group, it is suitable to apply Bonferroni correction and consider significant results when *p* < 0.017. Regarding WM, the group effect analysis showed that only the BAS group (Z =  − 2.824; *p*-value = 0.005; r =  − 0.706) improved significantly. The AER+R and CON groups reported no significant differences. Concerning CF, only the AER+R group showed a marginally significant improvement (Z =  − 2.331 *p*-value = 0.02; r =  − 0.549). Lastly, the group effect analysis in IC showed that the BAS group improved significantly in both parts of the Stroop test [part 3 score: (Z =  − 2.728 *p*-value = 0.006; r =  − 0.682); interference score: (Z =  − 3.154 *p*-value = 0.002; r =  − 0.789)] and also significantly improved in the reaction time of the incongruent (Z =  − 2.43 *p*-value = 0.015; r =  − 0.608) parts of the flanker task. The AER+R group significantly improved in the reaction time for the congruent (Z =  − 2.765 p-value = 0.006; r =  − 0.652) and incongruent (Z =  − 2.43 *p*-value = 0.015; r =  − 0.573) parts of the flanker task. Finally, the CON group showed significantly worse performance in the accuracy of the flanker task in the congruent (Z =  − 2.388 *p*-value = 0.017; r =  − 0.597) and incongruent trials (Z =  − 2.942 *p*-value = 0.003; r =  − 0.736) but improved in the reaction time for the neutral part (Z =  − 2.534 *p*-value = 0.011; r =  − 0.634). A visual representation of these results is depicted in Fig. 3.

### Between groups

**Figure 4:**
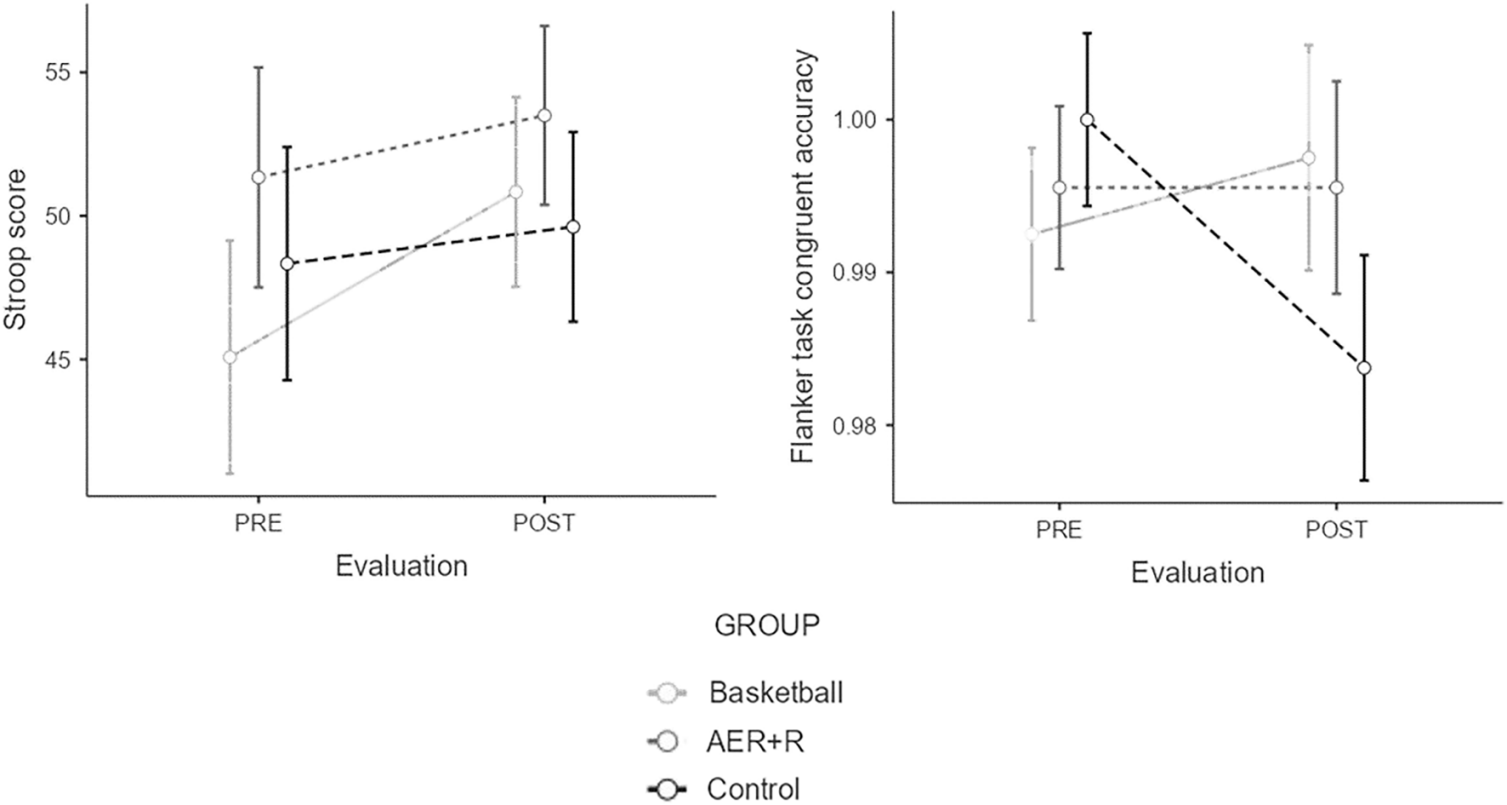
Significant differences between groups in Stroop test (left figure) and Flanker task (right image). Post-hoc analysis revealed that the basketball group improved significantly better than control group in the interference score of the Stroop test and the accuracy of the congruent trials in the flanker task.

A Kruskal–Wallis test revealed that only ΔIC showed significant results in the interference score of the Stroop Test (χ^2^ (2) = 5.646; *p* = 0.05). Bonferroni correction was applied in which a *p*-value < 0.017 was considered significant, but the post-hoc analysis did not reveal significant differences in the multiple comparison analysis, although a marginal result was observed as the BAS group had a better differential score compared to the CON group (Z =  − 2.299; *p*-value = 0.021; r =  − 0.406). In the flanker task, the accuracy of the congruent trials also revealed significant results (χ^2^ (2) = 9.482; *p* = 0.009); post-hoc analysis revealed again that the BAS group performed better than the CON group (Z =  − 2.783; *p*-value = 0.017; r =  − 0.492). These results are visually depicted in Fig. 4.

## Discussion

This study explored the differences among university students in the effects on EFs engaged in a four-month training PA program in uncertain environments with a high level of cognitive demands and those practicing PA without cognitive demands. One group practiced BAS, and the other followed an AER+R combined training program. A control group, which maintained low PA habits, was also included in the design. The BAS group showed improved IC and WM, while the AER+R group showed improved IC and CF. The control group showed deterioration in IC. Importantly, significant differences between groups were found only in IC, in line with the hypothesis.

### Working memory

WM was assessed through the Letter and Number Test of the WAIS battery. Paired sample tests revealed that the BAS group improved significantly, while both the AER+R and CON groups did not have significant differences. Considering that previous studies had observed that AER improves WM^4,16^, the finding that the AER+R group did not improve is surprising. Indeed, previous studies have shown that PA is associated with brain areas related to WM, and it has also been found that combined AER+R interventions improve WM significantly^4,8^. It could be that this incongruence may be due to differences in the training programs, such as PA type or intensity. For instance, the resistance training program in Quintero et al.^8^, in which they observed that combined exercise improved WM, consisted of working at 50–70% of one-repetition maximum, while our study also included isometric and explosive exercises.

Concerning the improvement in the BAS group, this result is similar to those of experimental studies performed in children that applied PA aimed at improving complex motor skills^34^. This adds more evidence observed in cross-sectional and longitudinal studies of the relationship between motor domain and WM, in which it is hypothesized that the neural substrates involved in both movement and cognitively complex tasks are associated with this EF, especially when there is a preparation time before the task (see Ludyga et al.^16^ for more details in proactive control); this might explain the differences between the BAS and AER+R groups, although ANOVA tests revealed no significant differences between the groups.

### Cognitive flexibility

The results revealed that only the AER+R group improved significantly, although not enough to yield significant differences between groups in the ANOVA analysis. This is surprising as it was hypothesized that the BAS group would improve significantly. According to Moen et al.^20^, goal-directed actions are the result of a comparison of the information stored in WM and the relevant experience that allows discrimination of what information is essential, followed by the application of IC in not reacting to information that could worsen the decision chosen. Players of a dynamic team sport, such as basketball, are constantly facing these situations^19^, so, again, it was expected that the BAS group would show significant improvement. However, the AER+R group was the only one to improve significantly. It has, however, been observed that regulating pace during a race or maintaining effort during an intense exercise requires maintaining and updating the objectives related to the exercise in WM^35^, so it seems that these physical tasks also have cognitive demands that could explain this result.

### Inhibition control

IC was assessed through two different tests: the Stroop test (inhibition response) and the Flanker task (perceptual inhibition). Paired sample tests showed that both the BAS and AER+R groups significantly improved in IC, while the CON showed significantly worse performance on the flanker task. This EF was the only one showing significant differences between groups in the ANOVA analysis, as the benefits achieved in the BAS group were significantly better than those of both the AER+R and CON groups.

These results are in concordance with studies observing that PA intervention improves EFs significantly^36^. For instance, the study of Alvarez-Bueno et al.^9^ showed an increment improvement in IC after an intervention of chronic exercise. In the present study, the CON group was asked to maintain their low PA habits, and it was the only group whose performance significantly deteriorated, while the two experimental groups, BAS and AER+R, improved in both IC tests, which is in line with the literature, in which IC shows significant improvements after chronic aerobic exercise interventions^9^. Indeed, in both groups, AER was present and linked to elevated levels of brain-derived neurotrophic factors (BDNF)^37^. However, in studies that included a combined AER+R intervention, despite showing greater improvement in IC, the effect size was somewhat smaller than ours^8^. According to Borst et al.^38^, combined physical interventions produce increased levels of IGF-1, which is related to BDNF in a different way and yields less improvement in IC than AER. Nevertheless, the effect size of the AER+R group was greater than that found in the literature, meaning that it is necessary to control the task-related factors and methodological interventions to achieve a consensus.

This EF was also the only one showing significant differences between groups, as the benefits achieved in the BAS group were significantly greater than those in both the AER+R and CON groups. These results are aligned with the reviews and meta-analyses showing inhibition as the EF most influenced by PA with cognitive engagement and reporting positive effects found in children and adults^9,16^. In fact, open-handed sports such as BAS can place higher demands on the individual’s inhibitory skills because they need to rapidly inhibit predominant responses due to the spontaneous and unexpected actions of other players on the court^39–41^. This supports the cognitive stimulation hypothesis, whereby interventions that include high levels of cognitive engagement and physical exertion are believed to have more substantial effects than physically demanding exercise with low cognitive engagement. It has also been shown in neuroimaging studies that performing open and complex motor skills, which require deeper information processing relative to simpler patterns, generates more consistent neuroplasticity changes^22^. In this line, children who practice team sports tend to show greater development in the prefrontal areas. According to researchers, the cause could be in cooperation with teammates, which requires greater cognitive complexity^42^. Therefore, considering that the BAS participants were continuously stimulated with these cognitive abilities, the intervention may have promoted greater stimulation, which caused bigger benefits in IC compared with the other groups.

This result is not in line, however, with the recent meta-analysis done by Ludyga et al.^16^, who found no differences between mixed (PA with cognitive engagement) and endurance interventions. However, this observation has to be taken with caution, because only four studies among those included in the meta-analysis compared mixed exercise and endurance exercise, and the samples in three of them were composed of older adults^35,43^, while one was with preadolescents^44^. None were in young adults, as in our study. In any case, their results are not very enlightening; in none of the three studies carried out in older adults were differences between the interventions applied, although, in two of them, the training sessions improved EFs^35,43^. In contrast, the study performed by Schmidt^44^ found only benefits in the mixed exercise group. This heterogeneity could be explained by the lack of control in the complexity of the tasks. As Antunes et al.^35^ stated, participants would need to exceed a minimum threshold of stimulation to facilitate cognitive processes and promote changes in the EFs. Indeed, after reviewing the programs that influence EFs, Diamond and Ling established that to find differences between the treatment and control groups, the tasks should require participants to use EF skills that are close to their limit. These demands affect both mixed and resistance exercise. In the study of Schmidt et al.^44^ and in ours, mixed exercises were designed to have a specific cognitive implementation.

### Strengths, limitations, and practical applications

Although most of the variables were controlled, some aspects of the present study could be improved. Specifically, considering that the BAS group was submitted to training with constant attentional and decisional demands, and the difficulty of the tasks gradually increased (taking into account the level of the participants in each moment of the intervention period), it would have been convenient to measure participants’ mental load at the end of each session. Two factors could have been responsible for the lack of stimulation: (1) the time per week dedicated to PA in this study was probably shorter than necessary; and (2) some EFs might not have improved because of the insufficient mental load presented in the sessions. Thus, future studies have to consider both an increment of the hours per week and measurement of each session’s perceived mental load. Note also that the final study sample did not correspond with the output of the power analysis, so the conclusions of this study should be interpreted with caution. Nevertheless, we hope that this study contributes to the understanding of the benefits of PA for EFs and highlights the need for future studies to include a PA training program in which participants have to pay attention to external stimuli and make decisions. Finally, concerning the cognitive evaluation, the risk of using only one task for EF could lead to an impurity problem.

In conclusion, following a four-month PA training program, two hours per week was enough to enhance EFs in university students with low PA habits. It was also observed that in the PA with cognitive engagement proposed in this study (i.e., playing basketball) the improvements of EFs, particular IC, were more evident. This combined dual-task training in which the individual performs exercise and cognitive tasks simultaneously is common in most open sports, so it could be of interest to include this type of practice in future cognitive studies.

## Data Availability

The datasets used and/or analyzed during the current study available from the corresponding author on reasonable request.
